# Involvement of Transforming Growth Factor Beta Family Genes in Gonadal Differentiation in Japanese Eel, *Anguilla japonica,* According to Sex-Related Gene Expressions

**DOI:** 10.3390/cells10113007

**Published:** 2021-11-04

**Authors:** Chien-Ju Lin, Shan-Ru Jeng, Zhen-Yuan Lei, Wen-Shiun Yueh, Sylvie Dufour, Guan-Chung Wu, Ching-Fong Chang

**Affiliations:** 1 Department of Aquaculture, National Pingtung University of Science and Technology, Pingtung 912, Taiwan; x747@g4e.npust.edu.tw; 2 Department of Aquaculture, National Kaohsiung University of Science and Technology, Kaohsiung 811, Taiwan; g73307955@gmail.com (Z.-Y.L.); yws@nkust.edu.tw (W.-S.Y.); 3 Laboratory Biology of Aquatic Organisms and Ecosystems (BOREA), Muséum National d’Histoire Naturelle, CNRS, IRD, Sorbonne Université, CEDEX 05, 75231 Paris, France; sylvie.dufour@mnhn.fr; 4 Center of Excellence for the Oceans, National Taiwan Ocean University, Keelung 202, Taiwan; 5 Department of Aquaculture, National Taiwan Ocean University, Keelung 202, Taiwan

**Keywords:** *amh*, *gsdf*, gonadal development, sex differentiation, fish, eel, *Anguilla*

## Abstract

The gonochoristic feature with environmental sex determination that occurs during the yellow stage in the eel provides an interesting model to investigate the mechanisms of gonadal development. We previously studied various sex-related genes during gonadal sex differentiation in Japanese eels. In the present study, the members of transforming growth factor beta (TGF-β) superfamily were investigated. Transcript levels of anti-Müllerian hormone, its receptor, gonadal soma-derived factor (*amh*, *amhr2*, and *gsdf*, respectively) measured by real-time polymerase chain reaction (qPCR) showed a strong sexual dimorphism. Transcripts were dominantly expressed in the testis, and their levels significantly increased with testicular differentiation. In contrast, the expressions of *amh*, *amhr2,* and *gsdf* transcripts were low in the ovary of E2-feminized female eels. In situ hybridization detected *gsdf* (but not *amh*) transcript signals in undifferentiated gonads. *amh* and *gsdf* signals were localized to Sertoli cells and had increased significantly with testicular differentiation. Weak *gsdf* and no *amh* signals were detected in early ovaries of E2-feminized female eels. Transcript levels of *amh* and *gsdf* (not *amhr2*) decreased during human chorionic gonadotropin (HCG)-induced spermatogenesis in males. This study suggests that *amh*, *amhr2,* and especially *gsdf* might be involved in the gene pathway regulating testicular differentiation of Japanese eels.

## 1. Introduction

The eel (*Anguilla* spp.) is a catadromous basal teleost with a remarkable migratory life cycle. Mature eels spawn in the ocean, and leptocephali larvae drift toward the coast and metamorphose into glass eels, which grow in continental habitats and then develop as yellow eels. After several years of growth phase in continental waters, the yellow eels undergo a prepubertal secondary metamorphosis and transform into silver eels [1]. The silver eels migrate downstream of rivers toward their spawning ground in the ocean to reproduce. Eels are gonochoristic species with gonadal differentiation occurring during the yellow eel phase [2]. Environmental factors, such as population density, temperature, pH, and social interactions, were proposed to prevail over genetic sex determination in eels [3,4]. Gonadal differentiation and development in eels were also suggested to be related to body size rather than age [2,5,6,7]. The gonochoristic feature with an environmental sex determination pattern and gonadal differentiation occurring during the yellow stage in eels provides an interesting model for investigating the mechanism of both gonadal sex differentiation and development.

Among vertebrates, most species are gonochoristic (sexually mature individuals are either male or female), and some are hermaphroditic (individuals with ovary and testis tissues in sequential or simultaneous mode) [8,9,10,11]. Sex determination and gonadal differentiation are crucial events during developmental processes in vertebrates [8]. Two main sex determination mechanisms, genetic and environmental (GSD and ESD, respectively), exist. In GSD species, gonadal sex is determined by master sex-determining genes and related transcription factors. For example, the gonads will develop into testes under control of the *sex-determining region of the Y chromosome* (*SRY*) gene in mammals [12] or of the *Doublesex- and Mab-3- related transcription factor-1* (*DMRT1)* gene in birds [13]. Among teleosts, a variety of master sex-determining genes has been evidenced, such as *DM-domain gene on the Y chromosome*/a duplicated copy of *dmrt1* in the sex-determining regions of the Y chromosome (*dmY*/*dmrt1bY*) in medaka (*Oryzias latipes*) [14,15], *sexually dimorphic on the Y chromosome* (*sdY*) in most salmonids [16], and *sry-related high mobility group-box gene 3 on the Y chromosome* (*sox3Y*) in the Indian ricefish, *O. dancena* [17]. Moreover, several *transforming growth factor beta* (TGF-β) family-associated master sex determining genes were also found in other teleosts, such as *Y chromosome-specific anti-Müllerian hormone* (*amhy*) in the Patagonian pejerrey, *Odontesthes hatcheri* [18], *amh type II receptor* (*amhr2*) in the tiger pufferfish, *Takifugu rubripes* [19] and *gonadal soma-derived factor on the Y chromosome* (*gsdfY*) in the Luzon ricefish, *O. luzonensis* [20].

In ESD species, the sex is not determined genetically but rather by environmental factors, such as temperature, pH, salinity, photoperiod, density, and/or social interactions [21]. A well-known example is the environmental sex determination in some turtles whose sex is determined by temperature and is called temperature-dependent sex determination (TSD) [22]. TSD in some amphibians has also been reported [23]. ESD is widespread in teleost fish; for example, temperature effects on sex differentiation were reported in Nile tilapia (*Oreochromis niloticus*) [24], pejerrey (*O. bonariensis*) [25], European sea bass (*Dicentrarchus labrax*) [26], and the *Apistogramma* genus [27]. Population density is suggested as the main environmental factor that could override genetic sex determination in eels [3,4,28].

Among vertebrates, teleost fish have the greatest diversity and plasticity in sex determination and gonadal differentiation. Their sex determination mechanisms belong to GSD, ESD, or both [21]. After the initiation of sex determination and the expressions of sex-specific genes during the process of gonadal differentiation in vertebrates, sex steroids (endogenous/exogenous sources of androgens and estrogens) play critical roles and take the control in masculinization and feminization of the gonads, in GSD or ESD, and in gonochoristic or hermaphroditic fish, including eels [8,11,21,29,30,31,32,33,34,35,36].

The process of sex determination and sex differentiation is regulated by a series of sex-related genes, and some crucial sex-related genes have been intensively studied in vertebrates including teleosts [37,38], for example, *dmrt1* and *sry-related high mobility group*-*box gene 9* (*sox9*), both of which encode transcription factors. Sox9 has been suggested to play an important role in the testicular development in medaka [39], zebrafish (*Danio rerio*) [40], mouse (*Mus musculus*), and chicken (*Gallus gallus*) [41]. Dmrt1 has been reported as a crucial regulator of male gonadogenesis and testicular differentiation and development, including spermatogenesis in teleosts [42,43,44,45,46]; furthermore, *dmrt1* is necessary for testis development by transcriptionally regulating *amh* in zebrafish [47]. Gonadal *cyp19a1* (aromatase) plays important roles in the ovarian differentiation [9,30,48,49]. In addition, *factor in the germline, alpha* (*figla*), which encodes a germ cell-specific basic helix-loop-helix transcription factor, is associated with ovary differentiation and development in vertebrates, including in teleosts [50,51,52].

Amh and Gsdf are members of the TGF-β superfamily, which play critical roles involving cell growth, cell differentiation, apoptosis, and cellular homeostasis; moreover, *amh*, its receptor *amhr2*, and *gsdf* were shown as sex determining genes in some teleost fish as described above. In mammals, AMH, also named Müllerian inhibiting substance (MIS), is expressed in Sertoli cells and is responsible for the regression of the Müllerian ducts and regulation of testicular differentiation of embryonic testes through binding to its receptor, AMHRII [53,54]. Although teleost fish lack a Müllerian duct, the *amh* orthologous gene was first identified from the testes of Japanese eel (*A. japonica*) and originally named *eel spermatogenesis related substance* 21 (*esrs21*). *Amh* transcripts were detected in Sertoli cells of immature testes but disappeared during spermatogenesis. Therefore, Amh was proposed as a spermatogenesis-preventing substance and was suggested to be involved in the regulation of germ cell development [55]. The sexually dimorphic with male specific expression pattern of *amh* was detected in several other teleosts during testicular differentiation (olive flounder, *Paralichthys olivaceus* [56], zebrafish [57], black porgy, *Acanthopagrus schlegelii* [58], and orange-spotted grouper, *Epinephelus coioides* [59]) or during early sexual maturation (Atlantic salmon parr, *Salmo salar* [60]). In contrast, the transcript level of *amhr2* was not sexually dimorphic during gonad development in medaka [61] and tiger pufferfish [19]. Gsdf was only found in teleost fish, implying that it may possess unique functions. The gene *gsdf* was first identified from the rainbow trout (*Oncorhynchus mykiss*) embryos with genital ridges. Gsdf protein was exclusively expressed in the genital ridge somatic cells surrounding the primordial germ cells (PGC) during embryogenesis; moreover, Gsdf was particularly located in the Sertoli cells surrounding the type-A spermatogonia of testis and in granulosa cells of ovaries during gonadal development in rainbow trout [62]. Therefore, Gsdf was thought to play an indispensable role in PGC and spermatogonia proliferation in rainbow trout. The importance of *gsdf* participating in early germ cell development and male sex differentiation was also reported in other teleost fish, for instance, medaka [63,64], Nile tilapia [65], olive flounder [66], the Chinese tongue sole, *Cynoglossus semilaevis* [67], and spotted scat, *Scatophagus argus* [68]. Furthermore, *gsdf* was determined as a downstream gene of *dmrt1* in the male pathway of Nile tilapia and spotted scat [68,69]. Gsdf was also suggested to be essential for testicular differentiation by inhibiting estrogen production in Nile tilapia [69].

Our previous studies on the Japanese eel reported the expression of some sex-specific genes (*vasa*, *cyp19a1*; female: *figla*, *sox3*; male: *dmrt1*, *sox9a, foxl3a, foxl3b*) during gonadal differentiation and development [6,70]. However, the molecular regulators involved in sex determination and differentiation in eels need further investigation. In this study, we analyzed the *amh*, *amhr2*, and *gsdf* expression patterns and the cellular distribution of *amh* and *gsdf* during testicular and E2-feminized ovarian differentiation and development of Japanese eels. This report elucidates the potential roles of the TGF-β superfamily members on gonadal differentiation and development in eels. We further propose a model of the expression profiles of sex-related genes during the process of gonadal differentiation in Japanese eels.

## 2. Materials and Methods

### 2.1. Animals

Four batches of eels were obtained for the experiments. The first batch (*n* = 1200, 300 elvers per tank) of elvers, with a mean body length of 9.54 ± 0.96 cm and a mean body weight of 0.88 ± 0.34 g, was used for E2-induced feminization. The second batch (*n* = 200, 100 elvers per tank) of elvers with a mean body length of 11.73 ± 0.31 cm and a mean body weight of 1.69 ± 0.12 g was used for cellular localization of *gsdf in situ* hybridization (ISH)/Vasa immunohistochemistry (IHC) studies. The third batch (3 female and 3 male eels) of yellow eels (2–3 years old; male eels, mean body length of 55.3 ± 1.2 cm, mean body weight of 305.0 ± 11.7 g; female eels, mean body length of 64.6 ± 1.5 cm, mean body weight of 409.3 ± 57.3 g) was used for cellular localization of *gsdf* ISH/Vasa IHC, and also for quantitative polymerase chain reaction (qPCR) of tissue distribution of *gsdf*, *amh*, and *amhr2* transcripts. The fourth batch (*n* = 9) of silver eels (3–4-year-old male eels, mean body length of 66.9 ± 3.5 cm, body weight of 461.4 ± 38.0 g) was used for human chorionic gonadotrophin (HCG) treatment to induce spermatogenesis.

All the fish were obtained from the commercial eel farms in Pingtung in southern Taiwan. The experimental fish were acclimated for at least one week for further experiments and analysis. The fish were cultured in indoor 2.5-ton tanks with aerated freshwater and then reared with natural light and temperature conditions at the culture station of the National Taiwan Ocean University (NTOU), Taiwan. The animal experiments were performed in an appropriate way in accordance with the animal experimentation procedure guidelines under the supervision of Drs. Chang, Wu, and Jeng under the authorization of the NTOU Institutional Animal Care and Use Committee (no. 104008).

### 2.2. E2-Induced Feminized Eels

Feminization of elvers was performed at the university culture station. Control (*n* = 600, 300 elvers per tank) and E2-treated fish (*n* = 600, 300 elvers per tank) were orally administered with the control diet and the E2-ontaining diet of 10 mg E2 (Sigma-Aldrich Corp., St. Louis, MO, USA) per kg of feed for 180 days (8 April 2015 to 13 October 2015). All the control elvers developed into male eels in our experimental conditions [6]. The gonadal samples were collected for qPCR of *gsdf*, *amh*, and *amhr2* transcripts. The cellular localization of *gsdf*, *amh*, and *figla* ISH/Vasa IHC studies was also conducted.

**Figure 1:**
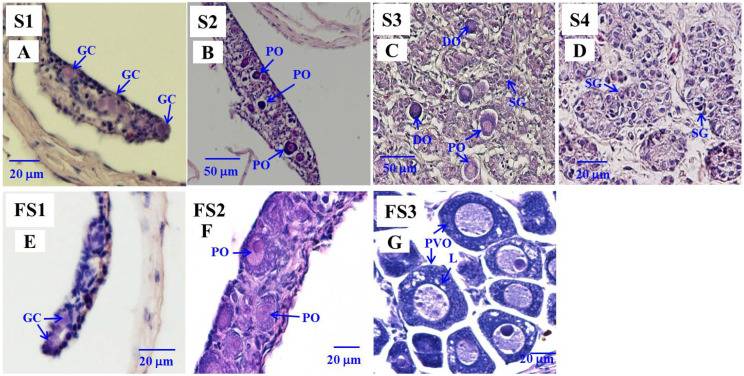
Gonad transverse sections, stained with hematoxylin and eosin of Japanese male eels at various stages (S1–S4) of testicular (**A**–**D**) differentiation and of estradiol-17β (E2)-feminized Japanese eels at various stages (FS1–FS3) of sexual differentiation (**E**–**G**). (**A**) S1 stage, undifferentiated gonad. (**B**) S2 stage, early differentiating gonad. (**C**) S3 stage, late differentiating gonad. (**D**) S4 stage, differentiated testis. (**E**) FS1 stage, undifferentiated gonad. (**F**) FS2 stage, differentiating ovary. (**G**) FS3 stage, differentiated ovary. GC, germ cell; PO, primary oocyte; DO, degenerating oocyte; SG, spermatogonia cell; PVO, pre-vitellogenic oocyte; L, lipid droplet.

Based on our previous histological examinations [6], the gonadal status in the control eels during testicular differentiation could be divided into undifferentiated (named S1, Figure 1A: only early germ cells were observed in the gonads), early, and late differentiating (early differentiating, named S2, Figure 1B: the gonads resembled ovaries and contained early germ cells, oogonia (OG), and primary oocytes (PO), late differentiating, named S3, Figure 1C: intersexual gonads exhibited a male-like testicular structure with OG, PO and degenerating oocytes (DO)), and differentiated (testis, named S4, Figure 1D: the testicular structures were formed). Based on our previous histological analysis [6], the E2-treated female eels could be divided into three stages: undifferentiated (named FS1, only early germ cells were observed in the gonads; Figure 1E), differentiating (named FS2, the gonads contained germ cells, OG and PO; Figure 1F), and differentiated eels (named FS3, OG, PO and pre-vitellogenic oocytes (PVO) were found in the gonads; Figure 1G).

### 2.3. HCG-Treated Male Eels

Nine 3–4-year-old male eels were randomly divided into two groups: (1) control (*n* = 5) and (2) HCG treatment (*n* = 4). Male eels were injected by HCG (Gona-5000 injection, China Chemical & Pharmaceutical Co. Ltd., Taipei, Taiwan) to induce testicular development. HCG treatment was conducted as described in our previous study [71]. Eels from the treatment group were given HCG (1 unit/g body weight/injection) consisting of one injection per week for six weeks. They were sacrificed four days after the final injection.

### 2.4. Sampling Procedures

Eels were anesthetized with 800 ppm of 2-phenoxyethanol before sacrifice. The biometric parameters of body weight and body length were measured. The gonadal tissue, or the body segment containing the gonadal tissue when the fish were too small to collect the gonadal tissue independently, was fixed overnight with 4% paraformaldehyde in phosphate-buffered saline (PBS, pH 7.4) for histological examinations and ISH/IHC analyses. For analyzing gene expressions, gonadal tissues were collected and stored at −80 °C until the quantitative real-time quantitative polymerase chain reaction (qPCR) analysis was performed.

### 2.5. Cloning of the Partial-Length amh, amhr2 and Full-Length gsdf cDNAs from Japanese Eel

The *amh, amhr2*, and *gsdf* cDNAs were cloned from the testis total RNA of Japanese eels by RT-PCR and rapid amplification of cDNA ends (RACE), following the manufacturer’s protocol (SMARTer^TM^ RACE cDNA Amplification Kit, Clontech, Tokyo, Japan). The specific primers used for cloning were designed according to the sequences from the NCBI nucleotide database and the Anguilla glass eel transcriptomic databases (http://molas.iis.sinica.edu.tw/4eels/index.html, accessed on 28 March 2017). After that, the resulting PCR products were cloned into the pGEM^®^-T Easy vector and sequenced.

### 2.6. Sequence Alignment and Phylogenetic Analysis

**Figure 2:**
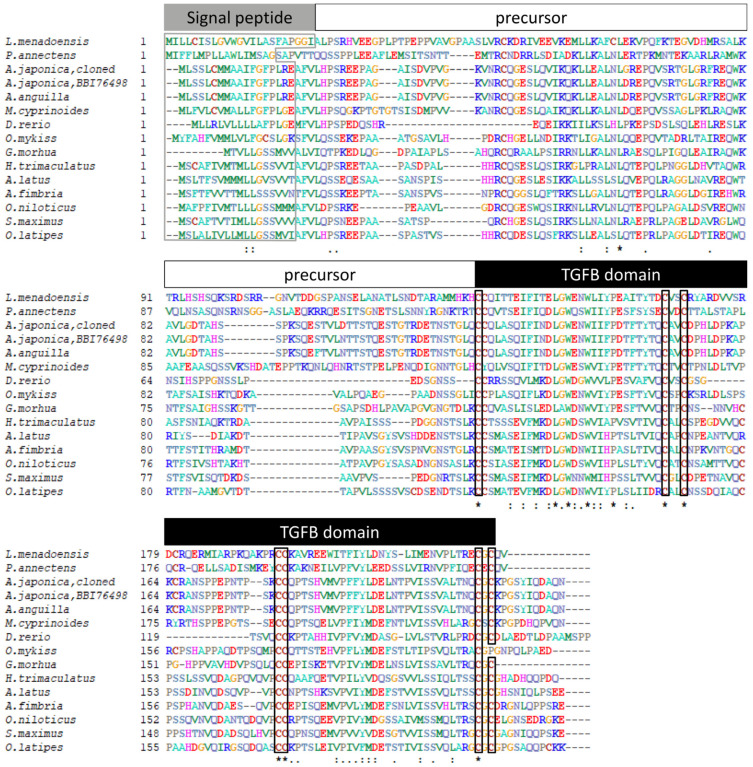
Alignment of the *gonadal soma derived factor* (Gsdf) amino acid sequences from Japanese eel and other fishes. The presumed signal peptide, precursor and transforming growth factor beta (TGF-β) domain are indicated. Signal peptides are boxed by gray line and conserved amino acids of the cysteine knot of TGF-β domain are boxed by black line. The symbols correspond to the identity percent of amino acid sequence comparison between fishes as asterisk to 100%, two spots to 80% and one spot to 60%, respectively. The sequences used are as follows: Menado coelacanth (*Latimeria menadoensis*, CCP19133), West African lungfish (*Protopterus annectens*, AWT24641), Japanese eel (*Anguilla japonica*, BBI76498), European eel (*A. anguilla*, XP35245734), Indo-Pacific tarpon (*Megalops cyprinoides*, XP36384635), zebrafish (*Danio rerio*, AFI98392), rainbow trout (*Oncorhynchus mykiss*, ABF48201), Atlantic cod (*Gadus morhua*, AGE15746), threespot wrasse (*Halichoeres trimaculatus*, BAM75186), yellowfin seabream (*Acanthopagrus latus*, AIW52571), sablefish (*Anoplopoma fimbria*, AGR33990), Nile tilapia (*Oreochromis. niloticus*, NP1266510), turbot (*Scophthalmus maximus*, AJO67894), and medaka (*Oryzias latipes*, NP1171213).

The Gsdf sequence cloned for this study was blasted to the NCBI database and identified. This Gsdf sequence from Japanese eel was further aligned with other fishes using the multiple sequence alignment tool (CLUSTALW, http://www.genome.jp/tools-bin/clustalw, accessed on 5 April 2021), and the identities were calculated. The presumed signal peptide sequences predicted by SignalP-5.0 (http://www.cbs.dtu.dk/services/SignalP-5.0/, accessed on 25 March 2021) are indicated in Figure 2.

For phylogenetic analysis, the TGF-β superfamily sequences of sarcopterygians and actinopterygians were retrieved from the NCBI database, and the accession numbers are indicated in Figure 2. The Amh, Bdnf, Gdf5, Gsdf, Inha, and Tgfb1 sequences in coelacanth (*Latimeria chalumnae*), tropical clawed frog (*Xenopus tropicalis*), chicken, mouse, human (*Homo sapiens*), sterlet (*Acipenser ruthenus*), Mississippi paddlefish (*Polyodon spathula*), spotted gar (*Lepisosteus oculatus*), alligator gar (*Atractosteus spatula*), Japanese eel, European eel (*A. anguilla*), Indo-Pacific tarpon (*Megalops cyprinoides*), Asian arowana (*Scleropages formosus*), *Paramormyrops kingsleyae*, rainbow trout, Nile tilapia, and medaka, along with the cloned Gsdf sequence in Japanese eel were used. The TGF-β domain sequences were determined in Amh, Gdf5, Gsdf, Inha, and Tgfb1 using the SMART system (http://smart.embl-heidelberg.de/, accessed on 26 March 2021) and applied in the phylogenetic analysis. In addition, the nerve growth factor (NGF) domain sequences determined in Bdnf were used as an outgroup. Multiple sequence alignments of TGF-β domain and NGF domain amino acid sequences were generated using MUSCLE, included in MEGA5.05 software (Pennsylvania State University, PA, USA) [72].

**Figure 3:**
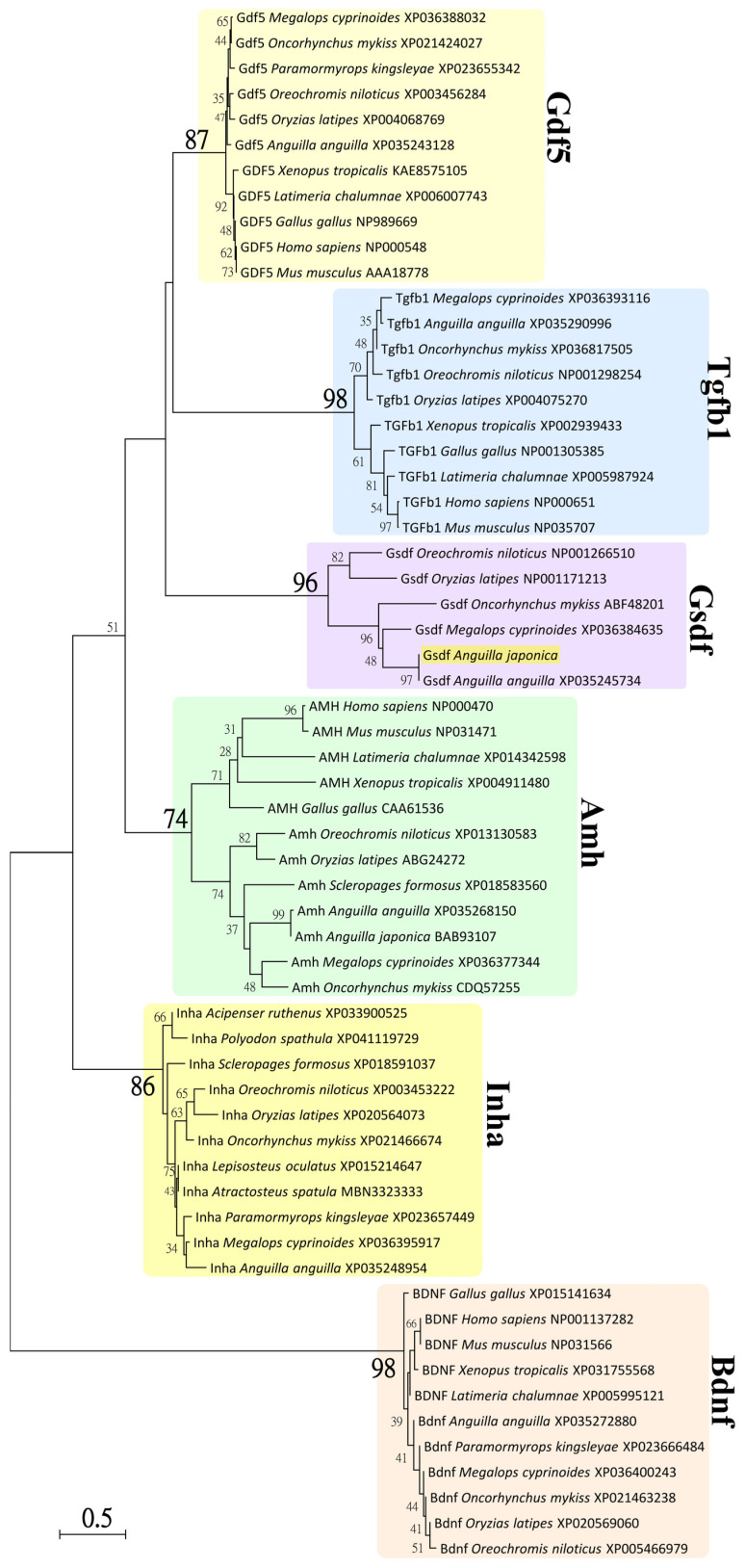
Neighbor-joining (NJ) phylogenetic tree of TGF-β superfamily amino acid sequences. Phylogenetic tree constructed based on 61 amino acid sequences of TGF-β domain or nerve growth factor (NGF) domain from TGF-β superfamily using the NJ method with 1000 bootstrap replicates. The number shown at each branch node indicates the bootstrap value as a %; only values and branching > 30% are shown. This tree was rooted using the sequence of NGF domain from Bdnf and shows major clades of Amh, Gdf5, Gsdf, Inha, and Tgfβ1 of TGF-β domain sequences. The cloned Gsdf sequence from Japanese eel clustered with the clade of Gsdf and is illustrated. The NCBI accession numbers are indicated and sequence were retrieved from the species of sarcopterygians: *Gallus gallus* (chicken), *Homo sapiens* (human), *L. chalumnae* (coelacanth), *Mus musculus* (mouse), *Xenopus tropicalis* (tropical clawed frog), and actinopterygians: *Acipenser ruthenus* (sterlet), *A. anguilla* (European eel), *A. japonica* (Japanese eel), *Atractosteus spatula* (alligator gar), *Lepisosteus oculatus* (spotted gar), *M. cyprinoides* (Indo-Pacific tarpon), *O. mykiss* (rainbow trout), *O. niloticus* (Nile tilapia), *O. latipes* (medaka), *Paramormyrops kingsleyae*, *Polyodon spathula* (Mississippi paddlefish), and *Scleropages formosus* (Asian arowana).

Phylogenetic analyses were conducted based on neighbor-joining (NJ) method with a best-fit Jones–Taylor–Thornton + Gamma (JTT+G) amino acid substitutions model in MEGA5.05 software [72]. Statistical support for the NJ tree was evaluated by 1000 bootstrapping replicates. A total of 61 amino acid sequences of representative species were used for phylogenetic analysis, including 12 Amh sequences (TGF-β domain), 11 Gdf5 sequences (TGF-β domain), 6 Gsdf sequences (TGF-β domain), 11 Inha sequences (TGF-β domain), 10 Tgfb1 sequences sequences (TGF-β domain), and 11 Bdnf sequences (NGF domain, used as an outgroup). The bootstrapping values < 30% are not shown in Figure 3.

### 2.7. Histology

Eel body segments or gonadal tissues were dehydrated through a graded methanol series and then embedded in paraffin. The transverse sections (4 μm) were stained with hematoxylin and eosin (H&E) staining to observe the gonadal status, which was classified via light microscopic examination. Our previous report was used as a reference to identify the characterization of gonadal histological structure [6].

### 2.8. Dual ISH and IHC

ISH and IHC staining were performed on the same slides to determine cellular localization and distribution of various genes. The methods from our previous study were modified slightly and followed [71]. IHC and specific antibody were utilized to label the Vasa protein, which was used as a germ cell marker based on our knowledge and previous study [6,43]. cDNA fragments of *amh* (1092 bp (base pairs), GenBank accession number: MZ905497), *gsdf* (964 bp, GenBank accession number: MZ905499), and *figla* (524 bp) were used to synthesize the RNA probe for ISH. Both sense and antisense riboprobes were synthesized with DIG RNA Labelling Mix (Roche, Indianapolis, IN) using T7 or SP6 RNA polymerases (Promega) by in vitro transcription. After deparaffinization with xylene, gonadal transverse paraffin sections (4 μm) were rehydrated through a graded series of ethanol (100–30%) and then in distilled water. Sections were then washed with 0.85% NaCl and PBS before post-fixation in 4% paraformaldehyde for 20 min. After washing in PBS, sections were treated for 5 min at 37 °C with proteinase K (2 mg/mL) diluted in PBS and then fixed for 15 min in 4% paraformaldehyde. Sections were treated with DNase I (5 U/mL) for 15 min. After washing in PBS, sections were rinsed twice in 2X standard saline citrate (SSC). Hybridization was performed at 68 °C overnight in a humidified chamber using 100 mL hybridization buffer (50% deionized formamide; 2X SSC; 5xDenhardt’s solution; 50 mg/mL of yeast tRNA; 4 mM ethylenediaminetetraacetic acid [EDTA]; 2.5% dextran sulfate) containing the digoxigenin (DIG)-labeled probe (3 mg/mL). After hybridization, slides were washed in 2X SSC at 65 °C, 2X SSC/50% formamide at 65 °C, 0.2X SSC, and 0.1X SSC at room temperature. Slides were washed in 100 mM Tris-HCl (pH 7.5) containing 150 mM NaCl for 10 min and then washed in the same buffer but containing 0.1% Triton and 0.5% skim milk powder, before being incubated overnight at room temperatures with anti-digoxigenin alkaline phosphatase Fab fragments (1:2000; Roche Pharma, Boulogne-Billancourt, France). The next day, slides were incubated with 2-hydroxy-3-naphtoic acid-2′-phenylanilide phosphate (HNPP)/Fast Red detection kit (Roche Pharma) according to manufacturer’s instructions. IHC steps were applied after ISH, first washing in PBS and sections were incubated with the guinea pig polyclonal antibodies against eel Vasa (1:1000). Sections were then immersed in the Alexa fluor goat anti-guinea pig (1:150; 488-conjugated; Invitrogen, Waltham, MA, USA) antibody. After washing away any excess antibodies, the slides were mounted with Vectashield mounting medium containing 4′,6-diamidino-2-phenylindole (DAPI) from Vector Laboratories (Burlingame, CA, USA) that visualize the cell nuclei. An epifluorescence microscope (Olympus Provis) equipped with a DP71 digital camera was used to examine and photograph sections. Digital images were processed with the Olympus Analysis Cell software (Olympus, Tokyo, Japan).

### 2.9. RNA Extraction and Reverse Transcription

Eel tissues were homogenized in Trizol reagent (Invitrogen), and total RNA was extracted following the manufacturer’s protocols. Total RNA was reverse-transcribed to obtain the first-strand cDNA using SuperScript^® III^ Reverse Transcriptase (Invitrogen) with oligo (dT)_12-18_ primers (Promega, Madison, WI, USA) according to the manufacturer’s protocol. The first-strand cDNA was used for cloning and qPCR.

### 2.10. Quantification of Gene Transcripts Using Real-Time Quantitative PCR

The qPCR analyses were conducted using the first-strand cDNA and the methods described in our previous study [6]. Primers for QPCR analysis were designed according to Primer Express version 3.0 (Applied Biosystems, Foster City, CA) and followed the design guideline (Table 1). The gene expression of Japanese eel *amh*, *amhr2*, *gsdf*, and *elongation factor 1 alpha* (*ef1a*) transcripts were analyzed. The cDNA fragments of each gene were cloned and used as standards and an internal control gene for qPCR analyses (*amh*, 1092 bp; *amhr2*, 1198 bp, GenBank accession number: MZ905498; *gsdf*, 964 bp; and *ef1a*, 1311 bp). Gene-specific primers were designed for qPCR and are described in Table 1. Quantification of gene expression in standards (plasmids with cDNA sequence) and samples was conducted concurrently using a 7300 Real-Time PCR System (Applied Biosystems, Foster City, CA, USA) with SYBR green I as a dsDNA minor-groove binding dye. Non-template controls were performed in conjunction with every application, and no amplification was found. The melting curve patterns indicated the amplification of a single amplicon for each gene. The slopes of the respective standard and sample curves of the log cDNA concentrations versus Ct (the calculated fractional cycle number at which the PCR-fluorescence product is detectable above a threshold) were −3.3–(−)3.5, indicating an amplification efficiency of 100% to 90%. Gene expression was analyzed using the 2^−∆∆Ct^ method while calibrating with an internal control *ef1a*. The highest transcript value in each comparison was defined as 1, and the relative gene expressions are presented.

### 2.11. Data Analysis

Transcript levels are presented as means ± standard error of mean (SEM). The values were analyzed using Student’s *t*-test to evaluate significant differences (*p* < 0.05) between groups. All statistical analyses were performed using SPSS10.0 (SPSS Inc., Chicago, IL, USA) for Windows.

## 3. Results

### 3.1. Sequence Analysis of Japanese Eel gsdf

The *gsdf* sequence with partial 5′UTR (untranslated region) of 292 bp and an open reading frame (ORF) of 669-bp that encoded 223 amino acids was obtained by cloning from testis total RNA (Figure S1). The signal peptide, TGF-β domain, and seven conserved cysteine residues are indicated in Figure 2. The ORF of *gsdf* was compared to the Gsdf sequence from GenBank (accession number: BBI76498). The identity of Japanese eel Gsdf between our cloned sequence (accession number: MZ905499) and BBI76498 was 99.10%, indicating 221 identical residues of 223 amino acids and two dissimilar residues in the precursor region. Additionally, this cloned ORF of Japanese eel was compared to European eel Gsdf (GenBank accession number: XP35245734). The identity between the two sequences was 98.65% (220/223) with a three-residue difference in the precursor region.

A multiple sequence alignment of Gsdf amino acid sequences of Japanese eel and other fishes was performed and is shown in Figure 2. The Gsdf protein sequences of Menado coelacanth (*L. menadoensis*, CCP19133), West African lungfish (*Protopterus annectens*, AWT24641), Japanese eel (BBI76498), European eel (XP35245734), Indo-Pacific tarpon (XP36384635), zebrafish (AFI98392), rainbow trout (ABF48201), Atlantic cod (*Gadus morhua*, AGE15746), threespot wrasse (*Halichoeres trimaculatus*, BAM75186), yellowfin seabream (*Acanthopagrus latus*, AIW52571), sablefish (*Anoplopoma fimbria*, AGR33990), Nile tilapia (NP1266510), turbot (*Scophthalmus maximus*, AJO67894), and medaka (NP1171213) were used.

### 3.2. Phylogenetic Analysis of Japanese Eel Gsdf

Phylogenetic analysis was used to estimate the evolutionary relationships between the Gsdf sequences of Japanese eel and TGF-β superfamily sequences of various vertebrates. Based on an alignment of amino acid sequences (Figure S2) and taking the NGF domain sequences determined in Bdnf as an outgroup, a phylogenetic tree was constructed using the neighbor-joining method with 1000 bootstrapping replicates in MEGA5.05 software [72].

As shown in Figure 3, the TGF-β domain sequences clustered into five major clades of Amh, Gdf5, Gsdf, Inha, and Tgfb1 sequences, respectively. The NGF domain sequences of Bdnf clustered into one clade as an outgroup. These clades were well supported by 74% to 98% bootstrap values. The clade of Gsdf sequences (96% bootstrap value) encompassed all teleost Gsdf sequences, including the cloned Gsdf sequence from Japanese eel, as illustrated. The phylogenetic analyses indicated that our cloned sequence of Japanese eel, just as the BBI76498 sequence from Japanese eel, is a Gsdf sequence and one member of TGF-β superfamily.

### 3.3. Tissue Distribution of amh, amhr2 and gsdf in Yellow Eels

**Figure 4:**
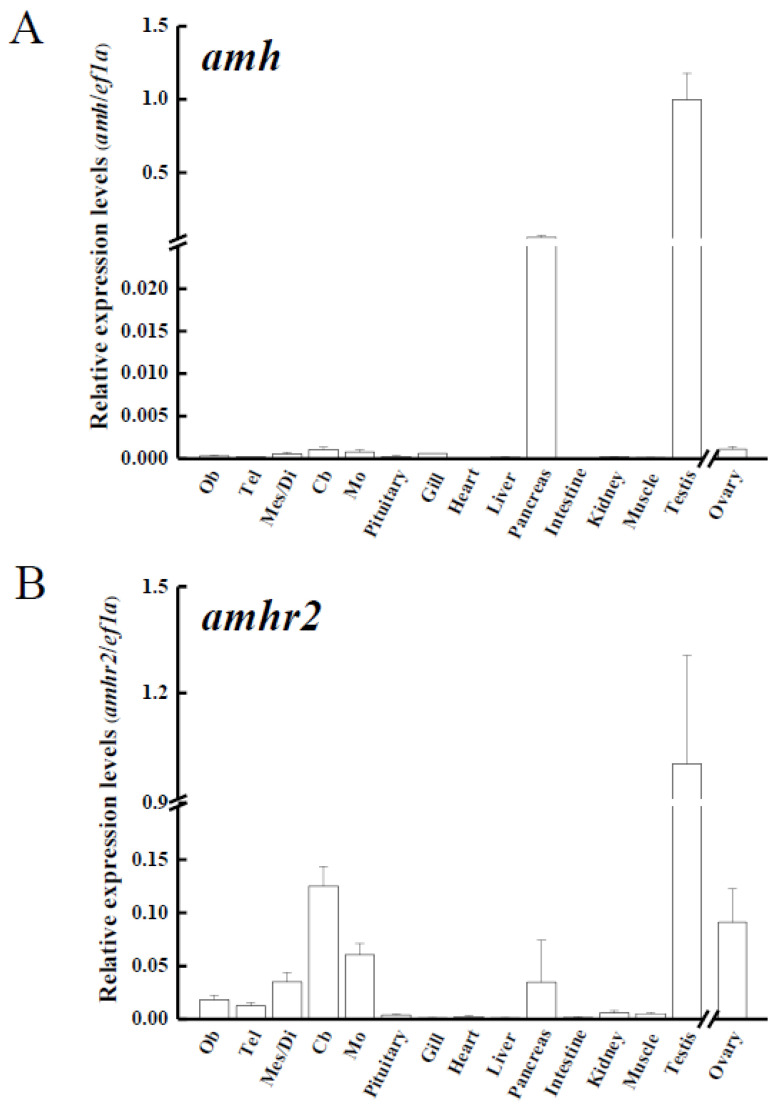

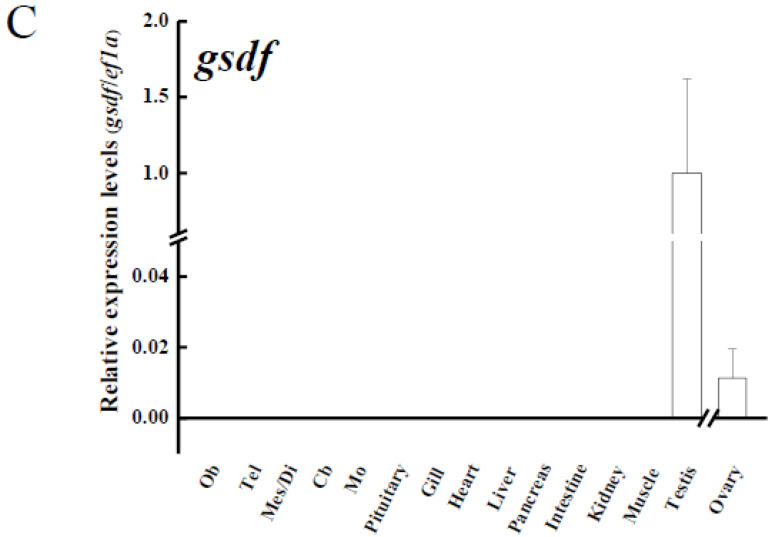
Transcript levels of (**A**) anti-Müllerian hormone (*amh*); (**B**) its receptor, *amhr2*, (**C**) gonadal soma-derived factor (*gsdf*) in various tissues of male Japanese eel and ovary of female Japanese eels (*n* = 3). Transcripts were measured by real-time polymerase chain reaction (qPCR). *ef1a* was used as an internal control gene. Data were expressed as means ± standard error of the mean (SEM). Ob: olfactory bulbs, Tel: telencephalon, Mes/Di: mes-/diencephalon, Cb: corpus cerebellum, Mo: medulla oblongata.

Various tissues from yellow male eels (*n* = 3) and ovaries from yellow female eels (*n* = 3) were used to analyze *amh*, *amhr2*, and *gsdf* transcripts in the Japanese eel. The transcripts of *amh* and *amhr2* (Figure 4A,B, respectively) were predominantly expressed in the testis, whereas slight expression of these transcripts were observed in the ovaries. The expressions of *amh* transcripts were also expressed in the pancreas and at low levels in other extragonadal tissues, including gills and different parts of the brain. Low levels of *amhr2* transcripts were also detected in extragonadal tissues (Figure 4A,B). *Gsdf* transcripts were exclusively expressed in the gonads, in which much higher levels in the testis rather than in the ovaries were found (Figure 4C).

### 3.4. Expression of amh, amhr2, and gsdf in the Gonads during Sex Differentiation in Eels

**Figure 5:**
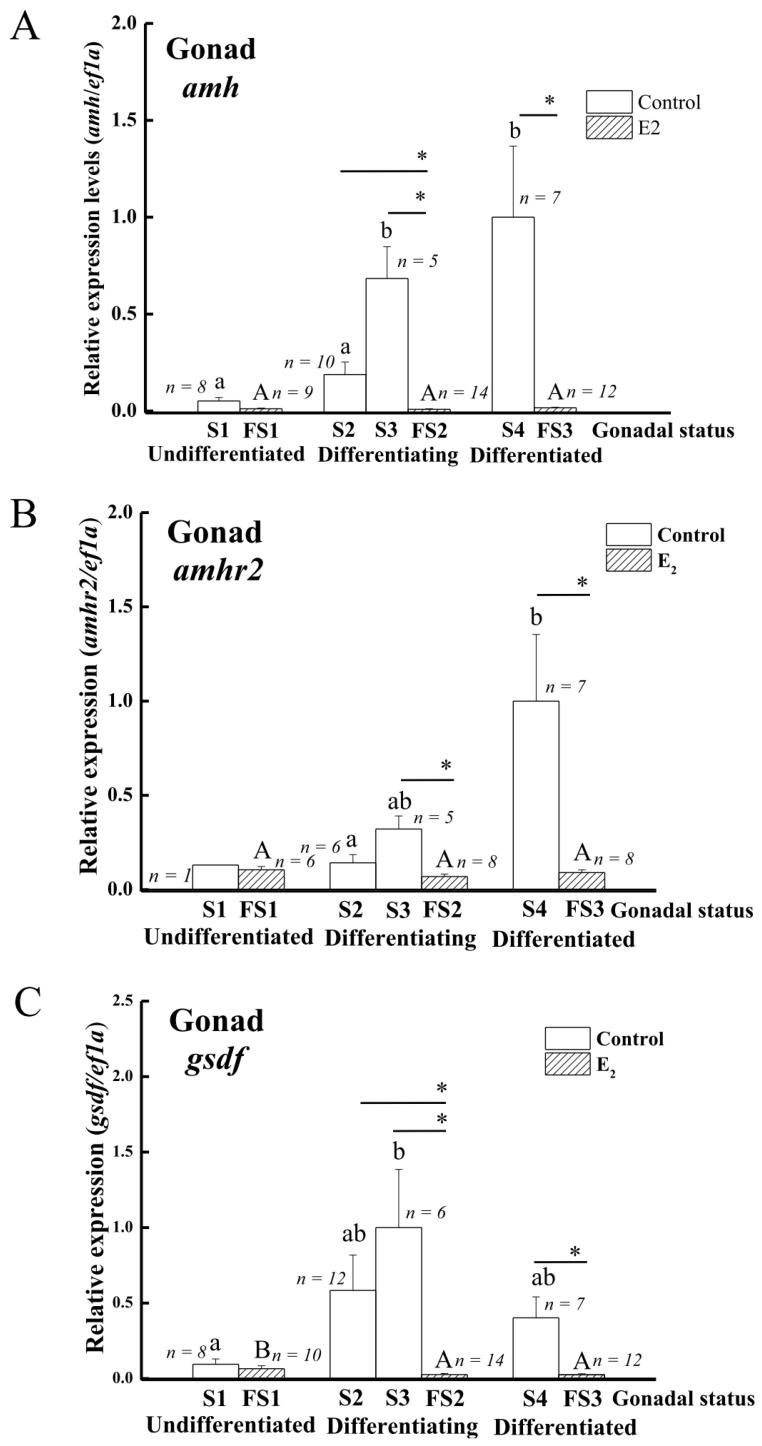
Expression profiles of sex-specific genes during sex differentiation in control and E2-induced feminized Japanese eels. (**A**) *amh*, (**B**) *amhr2*, (**C**) *gsdf*. Transcripts were measured by qPCR. *ef1a* was used as an internal control gene. Data were expressed as means ± SEM. The number in the figure showed the fish number in each group. Small and capital letters indicate significant differences (*p* < 0.05) among various gonadal status for the control groups and E2-induced feminized Japanese eels, respectively. Asterisks indicate significant differences (* *p* < 0.05) between the control eels and E2-induced feminized Japanese eels. For the control eels, S1, undifferentiated gonad; S2, early differentiating gonad; S3, late differentiating gonad; S4, differentiated testis. For the E2-induced eels, FS1, undifferentiated gonad (E2 treated group); FS2, differentiating ovary; FS3, differentiated ovary.

The transcript levels of *amh*, *amhr2*, and *gsdf* in the gonads were analyzed using qPCR to further elucidate gene expression profiles during sex differentiation in Japanese eels. The expressions of *amh* transcripts significantly increased in differentiating testis (S3) and differentiated testis (S4) compared to undifferentiated (S1) and early differentiating testis (S2) (Figure 5A). *Amhr2* transcripts significantly increased in differentiated testis (S4) compared to early differentiating testis (S2) (Figure 5B). The expressions of *gsdf* transcripts significantly increased in differentiating testis (S3) compared to undifferentiated gonads (Figure 5C). Furthermore, *amh*, *amhr2*, and *gsdf* transcripts demonstrated a strong sex-dimorphic expression (Figure 5A–C). The transcripts of *amh, amhr2,* and *gsdf* were maintained at low levels throughout ovarian differentiation in E2-feminized Japanese eels compared to their strong increase in control males during testis differentiation (Figure 5A–C). Low *gsdf* transcript levels decreased even further in the ovaries during differentiation (FS2, FS3) as compared to the undifferentiated stage (FS1), as shown in Figure 5C.

### 3.5. Expression of amh, amhr2 and gsdf Genes in the Gonads during Eel Spermatogenesis

**Figure 6:**
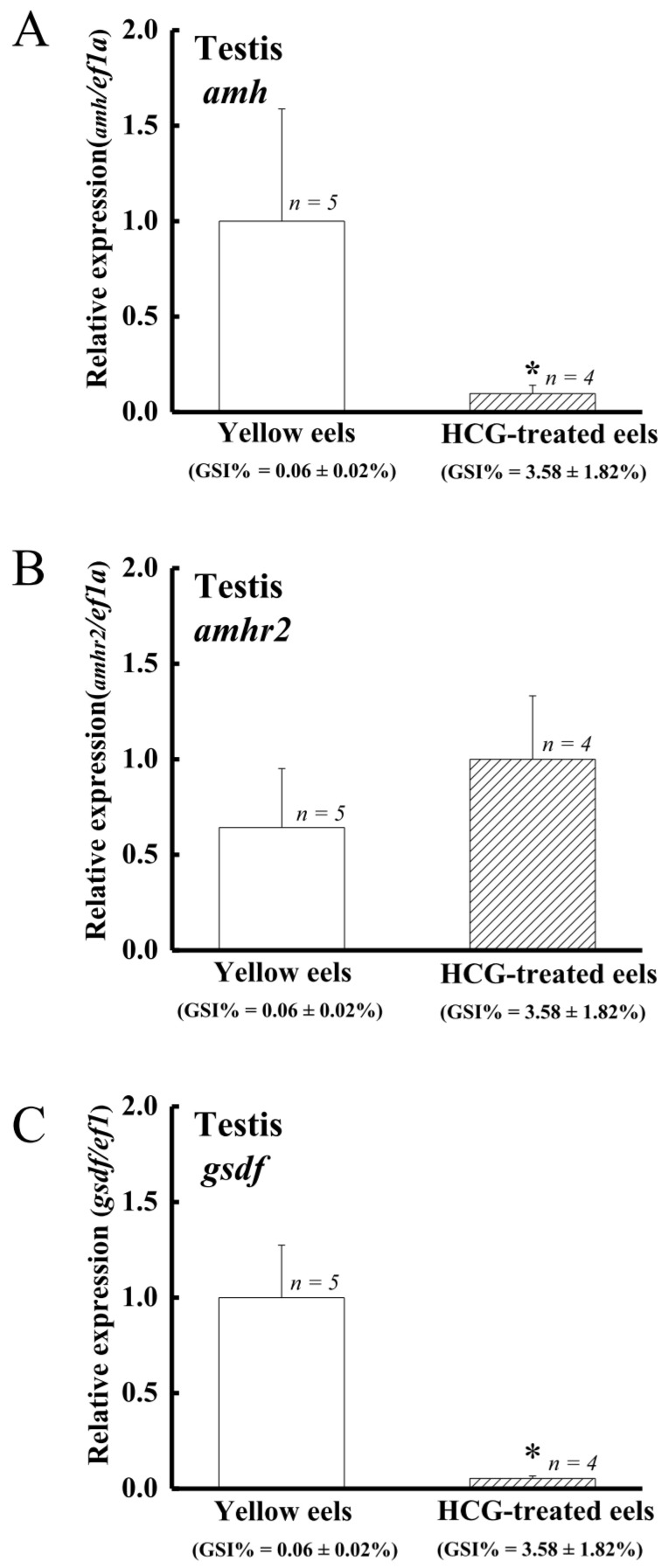
Profiles of (**A**) *amh*, (**B**) *amhr2*, and (**C**) *gsdf* expression in the control and HCG-treated male Japanese eels. Transcripts were measured by qPCR. *ef1a* was used as an internal control gene. Data were expressed as means ± SEM. Asterisks indicate significant differences (* *p* < 0.05) between the control male eels and HCG-treated male Japanese eels.

Similar to the previous studies [43,73], HCG was shown to induce testis development and spermatogenesis in eels. The histological analyses showed that the testis sections of control eels only contained spermatogonia A and B. In contrast, the testis of HCG-treated eels contained spermatogonia A and B, spermatocytes, spermatids, and spermatozoa (data not shown). The gonadosomatic index (GSI %) was 0.06 ± 0.02% in the control group and 3.58 ± 1.82% in the HCG group. The *amh* and *gsdf* transcript levels in testis significantly decreased in the HCG-treated eels compared to the control eels (Figure 6A,C), while no significant difference was observed for *amhr2* transcript levels (Figure 6B).

### 3.6. Cellular Localization of the amh and gsdf Transcripts in Eel Gonads

#### 3.6.1. Cellular Localization of the *gsdf* Transcripts in the Testis of Yellow Eels

**Figure 7:**
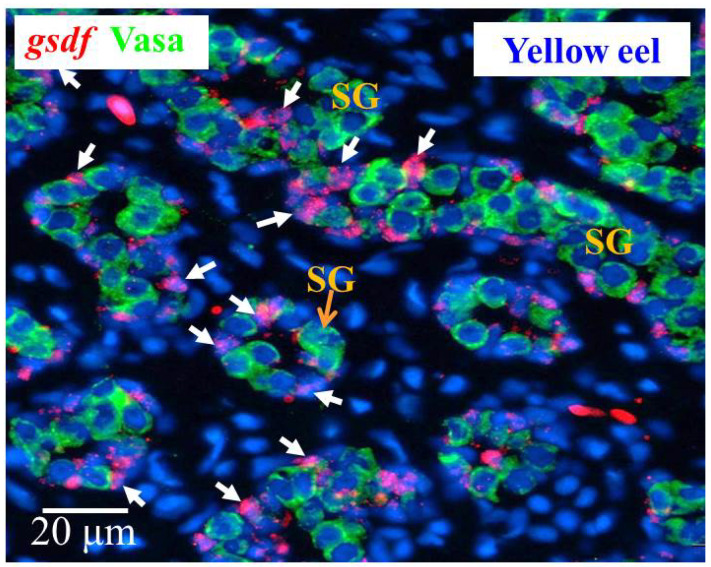
Cellular localization of *gsdf* transcripts and Vasa proteins in the yellow Japanese eel. *gsdf* transcripts (red) in the testis of the yellow eel as revealed by in situ hybridization (ISH) in Sertoli cells (white arrow). Vasa proteins (green) as revealed by immunohistochemistry (IHC) on the same sections. SG, spermatogonia cells.

Yellow male eels have differentiated testis, and the morphology of the cells in the gonad allows recognition of different cell types. We used yellow eel testis sections to determine the *gsdf* expressing cells. Our ISH results showed the *gsdf* transcript signals were specifically expressed in the Sertoli cells surrounding the spermatogonia (Figure 7).

#### 3.6.2. Cellular Localization of the *amh* and *gsdf* Transcripts during Testicular Differentiation in Eels

**Figure 8:**
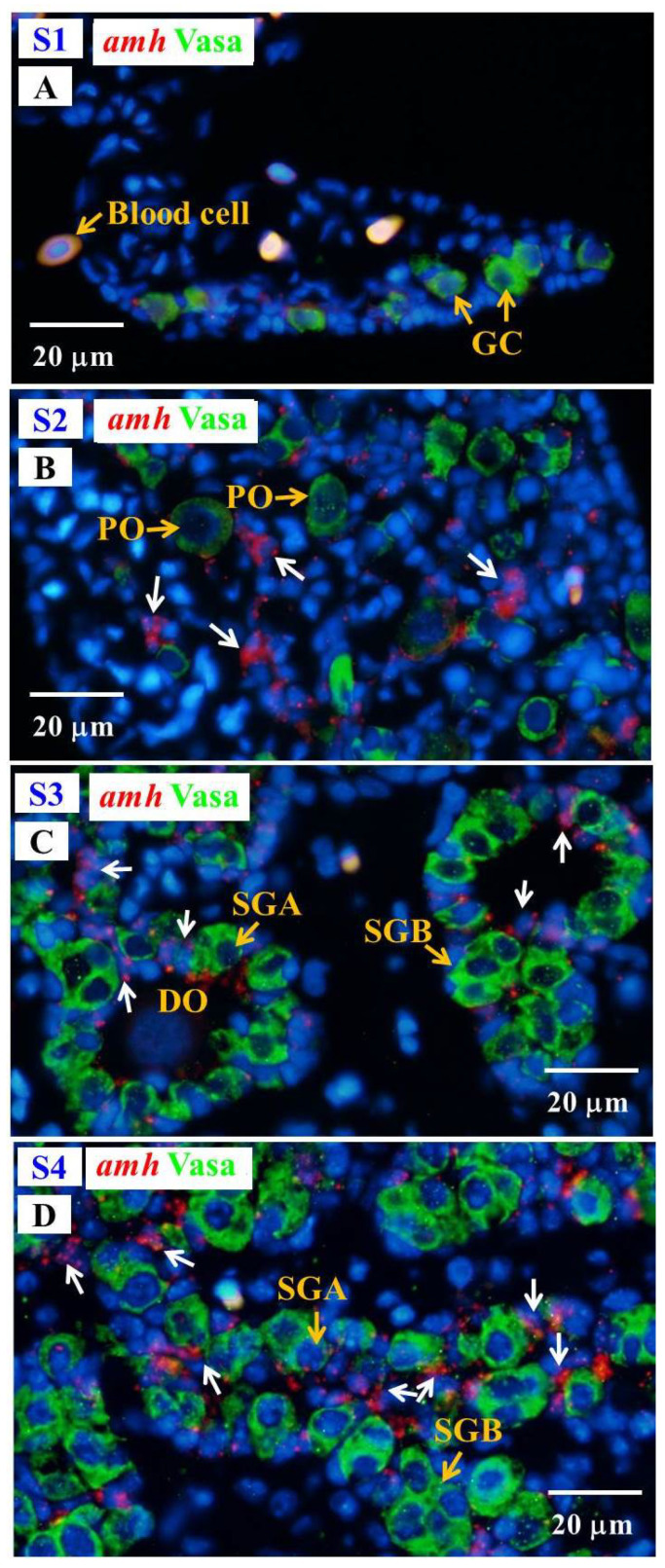
Gonad transverse sections, stained with ISH fluorescence for *amh* transcripts (red, white arrow) and IHC for the Vasa proteins (green) in male Japanese eel at various stages of testicular differentiation. (**A**) S1 stage, undifferentiated gonad. (**B**) S2 stage, early differentiating gonad. (**C**) S3 stage, late differentiating gonad. (**D**) S4 stage, differentiated testis. GC, germ cell; PO, primary oocyte; DO, degenerating oocyte; SGA, spermatogonia A cell; SGB, spermatogonia B cell.

Fuzzy and almost undetectable *amh* signals were found in the gonad at undifferentiated S1 stage; however, *amh* transcript signals were obviously observed in the gonads in the S2–S4 stages. The *amh* signals were detected in the Vasa-negative cells; moreover, the signals were specifically expressed in somatic cells surrounding the spermatogonia in late differentiating (S3 stage) and differentiated testis (S4 stage), and these somatic cells could be identified as Sertoli cells due to their position (Figure 8).

**Figure 9:**
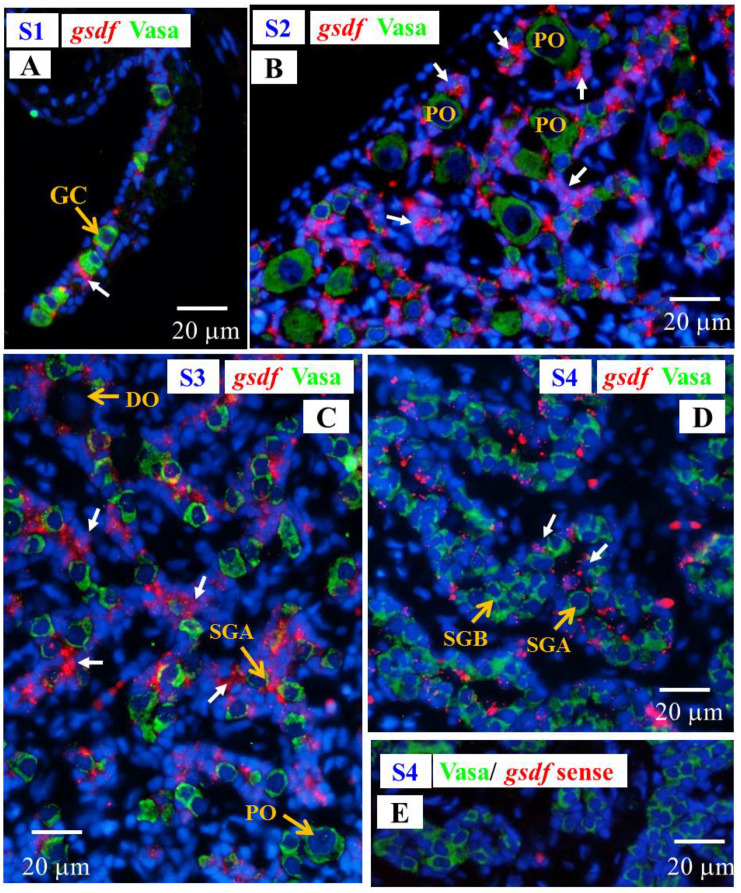
Gonad transverse sections, stained with ISH fluorescence for *gsdf* transcripts (red, white arrow) and IHC for the Vasa proteins (green) in male Japanese eel gonads at various stages of testicular differentiation. (**A**) S1 stage, undifferentiated gonad. (**B**) S2 stage, early differentiating gonad. (**C**) S3 stage, late differentiating gonad. (**D**) S4 stage, differentiated testis. (**E**) *gsdf* sense probe. GC, germ cell; PO, primary oocyte; DO, degenerating oocyte; SGA, spermatogonia A cell; SGB, spermatogonia B cell.

Signals of *gsdf* transcript were observed in the Vasa-negative somatic cells in the gonads at undifferentiated stage (S1); furthermore, intensive hybridization signals were detected in somatic cells of the gonads at differentiating stages (S2 and S3), while *gsdf* transcript signals became weaker in differentiated testis (S4) as compared with differentiating stage (Figure 9), in agreement with qPCR results (Section 3.4).

#### 3.6.3. Cellular Localization of the *amh*, *gsdf* and *fig1a* Transcripts during Ovarian Development in E2-Feminized Japanese Eels

**Figure 10:**
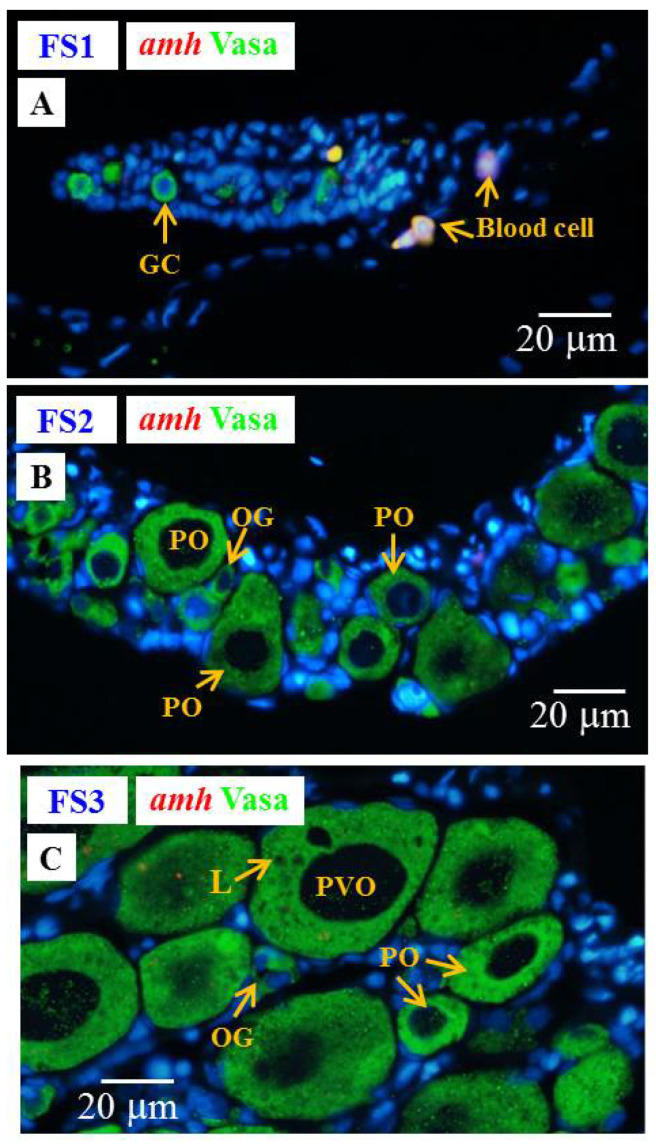
Gonad transverse sections, stained with ISH fluorescence for *amh* transcripts (red, no signal was observed in this figure) and IHC for the Vasa proteins (green) in E2-feminized Japanese eels at various stages of sexual differentiation. (**A**) FS1 stage, undifferentiated gonad. (**B**) FS2 stage, differentiating ovary. (**C**) FS3 stage, differentiated ovary. GC, germ cell; OG, oogonium; PO, primary oocyte; PVO, pre-vitellogenic oocyte; L, lipid droplet.

**Figure 11:**
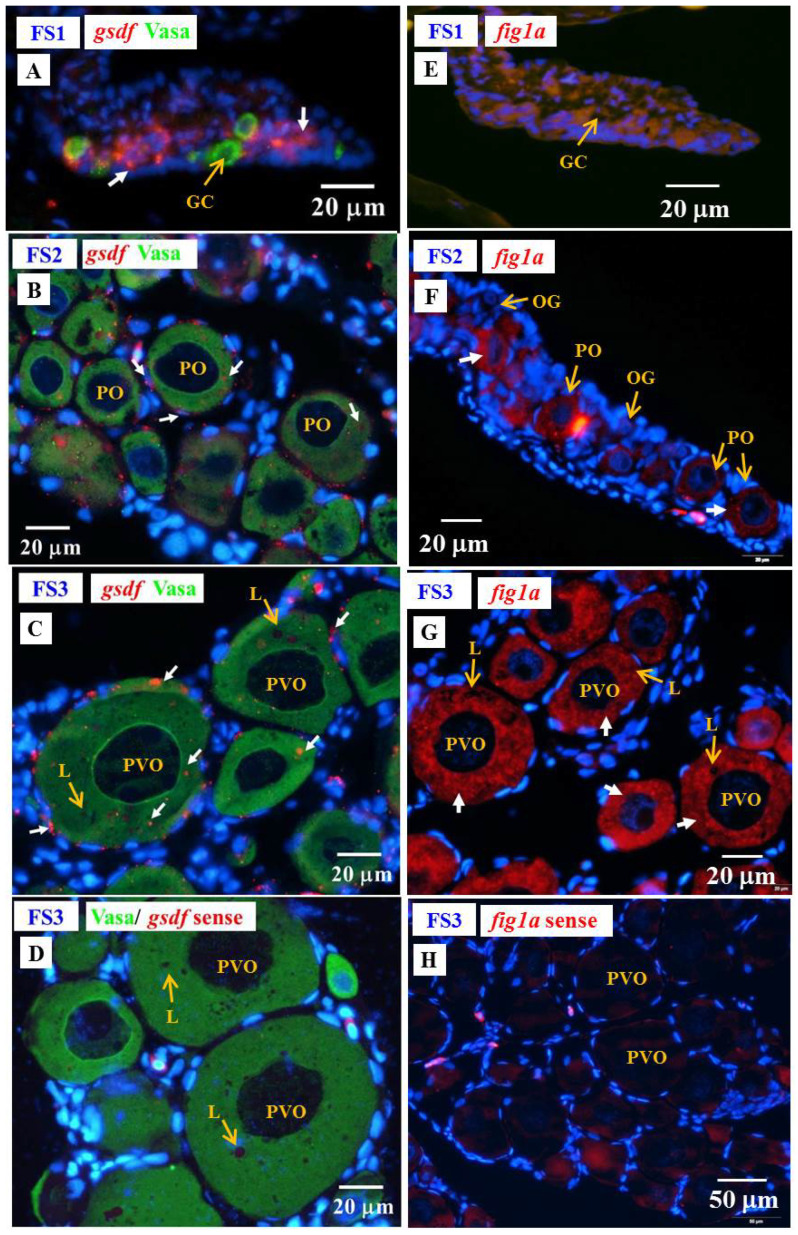
Gonad transverse sections, stained with ISH fluorescence for *gsdf* and *figla* transcripts (red staining, white arrow) and IHC for the Vasa proteins (green) gonads of E2-feminized Japanese eels at various stages of sexual differentiation. (**A**) *gsdf* at FS1 stage, undifferentiated gonad. (**B**) *gsdf* at FS2 stage, differentiating ovary. (**C**) *gsdf* at FS3 stage, differentiated ovary. (**D**) *gsdf* sense probe. (**E**) *figla* at FS1 stage. (**F**) *fig1a* at FS2 stage. (**G**) *fig1a* at FS3 stage. (**H**) *fig1a* sense probe. GC, germ cell; OG, oogonium; PO, primary oocyte; PVO, pre-vitellogenic oocyte; L, lipid droplet.

Low levels of *amh* and *gsdf* transcripts were measured by qPCR in the ovary of E2-feminized Japanese eels (Section 3.4). ISH did not show any *amh* signal in the gonads of E2-feminized Japanese eels (Figure 10), while *gsdf* transcript signals could be detected (Figure 11A–D). Positive *gsdf* signals were observed in the somatic cells of the gonad at the FS1 stage (Figure 11A); however, weak, but positive, *gsdf* signals were detected in the cytoplasmic of the primary oocytes and somatic cells surrounding the primary oocytes at the FS2 and FS3 stages (Figure 11B,C).

To follow the development of ovarian tissue, *fig1a* and *cyp19a1* were used. The *figla* transcripts significantly increased during ovarian development in E2-feminized eels as shown by qPCR in our previous study [6]. We further identified the cellular localization of *figla* in the ovaries by ISH in this study (Figure 11E–H). No *figla* transcript signals were detected in the gonad at the FS1 stage (Figure 11E), while strong *figla* signals were observed in the cytoplasmic of primary oocytes at the FS2 and FS3 stages (Figure 11F,G). No Cyp19a1 signals could be detected by IHC at any stage of E2-induced feminized eels and control eels (data not shown) due to its low expression in gonads [6]. Very low *cyp19a1* transcript levels were also detected by qPCR in the ovary of Japanese eels [6].

## 4. Discussion

### 4.1. Characterization of the gsdf cDNA and Phylogeny

The Japanese eel Gsdf protein contained 223 amino acids, including the typical TGF-β domain with seven conserved cysteine residues, similar to any other Gsdf sequences from fishes that we presented in Figure 2. The cysteine residues created a rigid structure, a unique cysteine-knot motif, which is related to intra-chain disulfide bonds or dimerization [74,75]. Two *gsdf* genes were found in Luzon ricefish [20] and European sea bass [76]. However, only one *gsdf* gene was found in the eel genome (Lin et al., unpublished data) and in most published teleost fish genomes. The phylogenetic analysis of the Gsdf conserved TGF-β domain obviously revealed that eel Gsdf with other teleost fish Gsdf formed a clade, and the clade was separated from other members of TGF-β superfamily. To our knowledge, this report is the first on concerning Japanese eel *gsdf* gene characterization and phylogeny, although *gsdf* was previously used as a Sertoli cells marker in Japanese eel for mRNA measurement [77], and *gsdf* expression pattern in gonads was analyzed using qPCR [77,78].

### 4.2. amh, amhr2 and gsdf Are Dominantly Expressed in the Testis of Japanese Eel

The members of TGF-β superfamily (*amh*/*amhr2*/*gsdf*), such as *amhy* in Patagonian pejerrey [18] and Nile tilapia [79,80], *amhr2* in tiger pufferfish [19], and *gsdfY* in Luzon ricefish [20], have been implicated in playing crucial roles in gonadal determination. The genes of the TGF-β superfamily were shown to also be involved in gonadal differentiation and development in some teleost fish [68,81]. The *amh* of fish was first found in Japanese eel [55], and recent studies have shown that the expression levels of *amh* and *gsdf* are significantly higher in testes than in ovaries of E2-treated Japanese eels [78]. The *amh* and *gsdf* transcripts of European eels were shown to be significantly higher in intersexual and male gonads than in females [82]. However, the roles of *amh*, *amhr2*, and *gsdf* in gonadal differentiation and/or development were still unclear at that point. Similar to the most studied teleost fish, our present study in Japanese eel showed that *amh* and *amhr2* transcripts were dominantly expressed in testis. However, very low expression levels of *amh* and *amhr2* in the ovaries and extragonadal tissues were also detected. This result is agreement with data obtained from some other teleost fish, such as *amh* and *amhr2* in Nile tilapia [83,84,85], *amh* in pejerrey [86], European sea bass [87], and Atlantic cod [88]. Due to the wide tissue distribution of *amh* in Nile tilapia, it has been proposed that *amh* may have multiple functions not restricted only to the gonads [81,85], which is also suggested according to our findings. In contrast, the expression levels of *amh* and *amhr2* in medaka were not sexually dimorphic with no extragonadal expression, and medaka *amh* and *amhr2* are thus suggested to be related to gonad formation and maintenance in both sexes [61]. Our study showed that *gsdf* expression in the Japanese eel was restricted to the gonads with a much higher expression in the testis than in the ovary. The same results were also found in rainbow trout [62], medaka [64], Nile tilapia [65], Chinese tongue sole [67], and spotted scat [68].

### 4.3. amh/amhr2 Might Play Important Roles in Testicular Differentiation in Japanese Eel

An increase in gonadal *amh* expression levels during male sex differentiation was reported in rainbow trout, Nile tilapia, pejerrey, black porgy, Atlantic cod, and zebrafish [81]. The expression levels of both *dmrt1* and *amh* showed a rapid increase followed by a decrease in the expression of *foxl2* and *cyp19a1a* during the key stage of the sex differentiation in temperature-masculinized XX females Nile tilapia; therefore, *dmrt1* and/or *amh* have been proposed as the modulator(s) of *foxl2* and/or *cyp19a1a* downregulation in temperature-induced testis development in Nile tilapia [89]. Furthermore, higher expression of *amh* was found at male inducing temperatures than at female inducing temperatures during the gonadal differentiation period in pejerrey [90] and Southern flounder (*Paralichthys lethostigma*) [91]. Male zebrafish have ovary-like gonads before the ovary-to-testis transformation [57]. Low *amh* transcript signals in undifferentiated gonads of zebrafish larvae were detected by ISH, and the *amh* signals gradually increased during the ovary-to-testis transformation. Moreover, intensive signals of *amh* were detected in testis, while no signal was found in the ovary in differentiated juvenile zebrafish [57]. Our present study showed the gonadal morphology of early male differentiating gonad of Japanese eel was similar to that of juvenile zebrafish and exhibited an ovary-like gonad (Figure 1). An increase in *amh* transcript levels was shown during testicular differentiation in Japanese eel, similarly to zebrafish. Our results suggest an important role of *amh* in testicular differentiation of eels. In addition, Amh was suggested to regulate germ cell proliferation, a function that might be conserved in fish [81]. For example, *amh* was suggested to be required for germ cell proliferation during early gonadal differentiation in madaka [92]. Overexpression of Amh by *amh*-plasmid feeding could stimulate the spermatogonia proliferation and testicular development in undifferentiated, orange-spotted groupers [93]. In contrast, Amh could inhibit spermatogonial proliferation during spermatogenesis in Japanese eels [55].

Amh function via binding to AMHR2 was demonstrated in mammals [94]. Amhr2 and its ligand Amh have also been identified in some teleost fish [81], except in zebrafish, which possess the *amh* gene, but the *amhr2* gene was not found in the genome [95]. Black porgy *amhr2* transcripts concurrently increased with its ligand during testicular differentiation; moreover, both *amh* and *amhr2* transcripts were expressed in the somatic cells surrounding the spermatogonia [58]. Double ISH showed that medaka *amh* and *amhr2* were mainly co-localized in the gonadal somatic cells in both sexes during early gonadal sex differentiation, and the Amh/Amhr2 signals might be related to the regulation of germ cell proliferation and follicular development [92,96,97]. Furthermore, co-localization of *amh* and *amhr2* revealed that *amh* acts through the autocrine/paracrine regulatory system [92,96,97]. In the present study, we found that the expression patterns of *amhr2* were similar to its ligand during testicular differentiation; therefore, *amhr2* might also play a vital role during testicular differentiation in Japanese eels. The *amhr2* expressing cells in eel have not yet been identified. Therefore, further evaluation of the cellular localization of *amhr2* in eel gonads will be the next important task.

### 4.4. gsdf Is Expressed in Sertoli Cells and Might Play an Indispensable Role in Testicular Differentiation in Japanese Eel

Gsdf was reported to play a critical role in male sex determination and/or differentiation in teleost fish; moreover, Gsdf might accomplish its function by inhibiting estrogen synthesis [67,69]. Gsdf expression in Sertoli cells has been indicated in many teleost fish, such as rainbow trout [62], Nile tilapia [65], medaka [63], olive flounder [66], Chinese tongue sole [67], spotted scat [68], Atlantic salmon [98], and orange-spotted grouper [99]. The *gsdf* of olive flounder was expressed in somatic cells prior to the occurrence of gonadal differentiation, and the expression then quickly increased when testis differentiation was initiated. Therefore, *gsdf* was proposed to play important roles on promoting testis differentiation and early germ cell development [66]. Furthermore, *gsdf* was distinctly and primarily expressed in XY undifferentiated gonads of Nile tilapia before any other testis-differentiation-related gene was detected; overexpression of *gsdf* in XX Nile tilapia demonstrated that *gsdf* was sufficient to induce the male sex differentiation pathway, and consequently, *gsdf* was suggested to play a critical role in male determination and/or differentiation of Nile tilapia [65]. Gsdf was shown to suppress the proliferation activities of oogonia in orange-spotted grouper [99]. We found *gsdf*-positive signals in the Sertoli cells surrounding the spermatogonia in yellow eels. This result in the eel agrees with other teleost fish as we described above. Although *gsdf* was used as a Sertoli cell marker for mRNA measurement using qPCR in Japanese eels [77], our study was the first report indicating the cellular localization of *gsdf* in the eel gonads. The present study showed that the expression levels of *gsdf* increased significantly during testicular differentiation of Japanese eels; moreover, high *gsdf* expression levels and strong transcript signals were present at differentiating stages (S2 and S3). Interestingly, the *gsdf* expression pattern was different from those of *amh* and *amhr2* with the highest expression levels of gonadal *amh* and *amhr2* being found at differentiated stage. Furthermore, the *gsdf* transcript signals could be distinctly observed in somatic cells surrounding the germ cells in undifferentiated gonads, and the signals were detected earlier than *amh* in Japanese eels. Therefore, our finding in the Japanese eel reinforced the importance of *gsdf* articipation in male determination and/or differentiation in teleost fish.

### 4.5. The Expression of amh and gsdf but Not amhr2 Is Suppressed by Gonadotropin

Amh was first regarded as a spermatogenesis-preventing substance in Japanese eel [55]; furthermore, Amh was reported to have inhibiting effects on germ cell development and steroidogenesis in zebrafish [100] and medaka [97,101]. Therefore, down-regulation of Amh might be necessary for the effect of gonadotrophins on maturation [81]. Previous studies showed the expression of *amh* decreased during spawning season in male teleost fish [60,102,103]. The expression of testicular *amh* was inhibited by HCG injection in black porgy [58] and European sea bass [104]. Our present data also showed the *amh* transcripts in testis significantly decreased in HCG-treated male eels in agreement with a previous report in the Japanese eel [55]. Curiously, the expression level of testis *amhr2* was not significantly inhibited by HCG in the present study. The asynchronous expressions of *amh* and *amhr2* in male eels indicate that gonadotropins might have different regulatory functions toward *amh*/*amhr2*.

A previous study indicated that *gsdf* expression levels were reduced progressively during spermatogenesis in yellowfin seabream [105]. Our study also showed the testis *gsdf* transcripts significantly decreased in HCG-treated male Japanese eels. However, the data concerning the regulation and role of *gsdf* during fish spermatogenesis are still limited and need further investigation.

### 4.6. The Expressions of amh, amhr2, and gsdf in the Ovary Were Inhibited in E2-Induced Feminized Japanese Eels

The robust male-biased expressions of *amh*, *amhr2*, and *gsdf* have been extensively reported, as these genes are considered pivotal factors in male sex determination and/or differentiation in teleost fish. Conversely, our data showed the transcripts of *amh* and *amhr2* were maintained at low levels during ovarian differentiation and development in E2-induced feminized eels. A recent study on the Japanese eel also reported a similar result. The transcript levels of *amh* were lower in the undifferentiated gonads of E2-treated eels compared with controls (males) [78]. These results suggest that E2 inhibited *amh*/*amhr2* involvement in testicular differentiation and induced the ovarian formation in eels. The administration of E2 to undifferentiated black porgy led to low expression of *amh*, and the expression of *amh* remained at low level in E2-induced ovarian tissue [58]. EE2 (17α-ethinylestradiol) concentration levels in the environment range from <0.20 ng/L to 34.00 ng/L in water, as previously reported [106]. Exposure of EE2 at environmentally relevant concentrations (0.5 and 5 ng/L) to zebrafish during early life had a suppressive effect on the expression of *amh* and caused subsequent disruption of male gonadal development [107]. However, an asynchronous expression of *amh* and *amhr2* was shown in black porgy [58]. The expression levels of *amhr2* significantly increased in the differentiated ovary of E2- and aromatase inhibitor-treated black porgy, and the *amhr2* expression levels showed no differences between the ovary and control testis [58]. Therefore, *amhr2* may be related to gonadal development in both sexes of black porgy [58].

Although *gsdf* transcripts/Gsdf proteins are dominantly expressed in Sertoli cells, *gsdf*/Gsdf were also found in the ovaries of teleost fish. A previous study indicated that rainbow trout Gsdf was present in ovarian granulosa cells [62]. In zebrafish, *gsdf* was located in somatic cells of bipotential gonads and ovarian granulosa cells [108,109]; moreover, the genes related to lipid metabolism, vitellogenesis, and steroid biosynthesis were downregulated in homozygous *gsdf* knockout mutant female zebrafish. Therefore, a role for *gsdf* in the regulation of ovarian follicle maturation and gene expressions for steroid biosynthesis and female fertility in zebrafish was proposed [109]. Gsdf was also found in the somatic cells surrounding the oogonia of the ovary in spotted scat, and possibly played a role in oogonia developmental regulation [68]. In addition, *gsdf* transcript signals were observed in the somatic cells and cytoplasm of oocytes in olive flounder; therefore, *gsdf* was proposed to participate in early germ cell development, such as proliferation and differentiation of oogonia in olive flounder [66]. Our study showed *gsdf* transcript signals in somatic cells of undifferentiated gonads of E2-ferminized eels, and faint signals were also detected in follicle cells and cytoplasm of oocytes of differentiating and differentiated ovary in E2-ferminized Japanese eels. Our qPCR data also indicated that *gsdf* transcripts remained at low levels and decreased significantly during ovarian differentiation/development in E2-ferminized Japanese eels. Our results imply that eel *gsdf* might be involved in the regulation of early germ cell development in both sexes, and E2 could inhibit the expression of *gsdf* and promote the feminization of Japanese eels. The suppressive effects of estrogen on the *gsdf* expression were also shown in several teleost fish, and a previous study concerning Japanese eel also showed *gsdf* transcripts were significantly lower in the undifferentiated gonads of the E2-treated eels than in the controls (males) [78]. Exogenous E2 could reduce the *gsdf* expression levels in genetic male medaka during embryonic development [64]. The transcripts of Sertoli cell-specific *gsdf* of mature medaka significantly decreased, and the testis-ova was first found after 14 days of treatment with estradiol benzoate [63]. Conversely, exposure of embryos of Javafish medaka, *Oryzias javanicus* (ZZ/ZW), to EE2 caused significant expression of an estrogen up-regulated gene, *Choriogenin-H*; however, the expression of *gsdf* in ZZ Javafish medaka fry was not suppressed, and most of the XX fry did not sexually reverse [110].

## 5. Conclusions

During our preparation of this paper, a certain degree of similarity to part of our results in Japanese eels was published in 2021 [78]. However, our study still provided further valuable information. (1) The expressions of TGF-β superfamily genes in the gonads were conducted from elvers, juvenile eels, to yellow eels including undifferentiated stage, differentiating stage, differentiated stage, and even up to pre-pubertal stage in male and female. (2) Our data, for the first time, demonstrated the cellular localization of TGF-β superfamily transcripts in eel gonads, and the transcript signals were strongly expressed in males and weak in females. (3) This study was a part of our series studies of gonadal sex differentiation in Japanese eels. We provided integrated information (in addition to TGF-β superfamily) for sex related genes profiles during sex differentiation and further development of Japanese eels.

**Figure 12:**
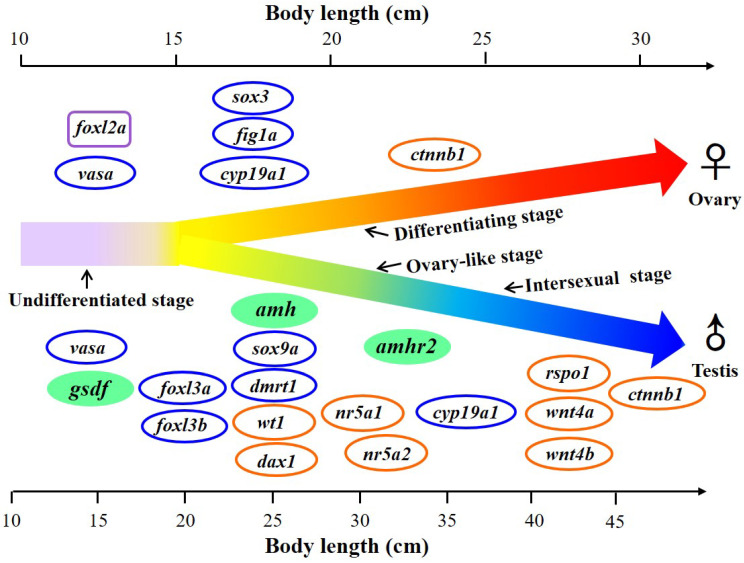
Proposed diagram of the expression of sex-related genes in the gonad during sex differentiation in Japanese eels. The gonadal differentiation occurs during the yellow eel stage at around 20–40 cm body length. E2 was applied to induce the feminization. Ovary-like and intersexual stages were found during the early testicular differentiation. Sex-related genes were sexually dimorphically expressed in the testicular and ovarian tissues. Sex-related genes with increased expression during the process of gonadal differentiation are indicated. The genes *gsdf*, *amh*, and *amhr2* were investigated in the present study and are marked as green. The genes previously published by Jeng et al. [6] and Wu et al. [69] are marked with blue circles, and the gene previously published by Inaba et al. [78] is marked with a purple square. The genes under investigation by Jeng et al. (unpublished data) are marked with orange circles.

Gonochoristic feature with environmental sex determination and gonadal differentiation occurred quite late during the yellow stage in Japanese eels (body length around 20–40 cm) and provides a unique model to study the mechanism of gonadal differentiation. A diagram of gene expression during gonadal sex development is proposed as a basis to elucidate the pathways of gonadal differentiation of Japanese eels (Figure 12). Genetic control for sex determination (the master regulator of male development) is highly diverse, while the downstream genetic pathways for sex differentiation is quite well conserved in vertebrates [8,21,111]. Sex-related genes were sexually dimorphic expressed in the testicular and ovarian tissues. Some sex-related genes (*vasa*, *figla*, *sox3*, *cyp19a1*, *dmrt1*, *sox9a*, *foxl3a*, *foxl3b*, *foxl2a*) were previously investigated during gonadal differentiation in Japanese eels [6,43,70,78]. We investigated the expression profiles of other sex-related genes according to the proposed model of gonadal sex development in Japanese eels (Figure 12). According to gene expression, our present data support that *amh*, *amhr2*, and especially *gsdf* might be involved in the gene pathway regulating testicular differentiation in Japanese eels.

## Figures and Tables

**Table 1:** Specific primers used for real-time quantitative polymerase chain reaction (PCR) analyses.

Gene	Sequences	Amplicon Size
*ef1a*	Sense: 5′-TGTGGGAGTCAACAAGATGGA-3′	58 bp
	Antisense: 5′-CTCAAAACGCTTCTGGCTGTA-3′	
*amh*	Sense: 5′-TCCTGGTCAGCACTGCGTATC-3′	60 bp
	Antisense: 5′-TCCCGCACCGACAGACA-3′	
*amhr2*	Sense: 5′-TCTGCATGGTGGTGGTTCCT-3′	66 bp
	Antisense: 5′-TGGATCTGTGGCACATGAAGA-3′	
*gsdf*	Sense: 5′-GAGCCAAACACCCCTTCAAA-3′	82 bp
	Antisense: 5′-GCGTGTTGAGCTCATCCAAGT-3	

## Data Availability

Data are contained within this article. Raw data are available on request from the corresponding authors.

## Supplementary Materials

The following are available online at https://www.mdpi.com/article/10.3390/cells10113007/s1, Figure S1: Nucleotide and deduced amino acid sequences of *A. japonica* Gsdf. Start codon is shown in box. Figure S2: Alignment of 50 TGF-β domain and 11 NGF domain amino acid sequences used in the phylogeny analysis (in Figure 3). Multiple alignment of TGF-β domain (transforming growth factor-beta family) of Amh, Gdf5, Gsdf, Inha and Tgfb1; and NGF domain (nerve growth factor) sequences of Bdnf. Conserved amino acids of the cysteine knot of TGF-β domain are boxed.
