# Supplementary material for: Involvement of Transforming Growth Factor Beta Family Genes in Gonadal Differentiation in Japanese Eel, Anguilla japonica, According to Sex-Related Gene Expressions
Source: Cells. 2021 Nov 4;10(11):3007. doi: 10.3390/cells10113007 (PMC8616510; doi:10.3390/cells10113007)
Supplement: Supplementary file 1 [file cells-10-03007-s001.zip › Figure S1.pdf]

```
AATCTCTCCCTCGTACTGTCTCACGCTTGCGGGACAGAAAAACAATGTG
AAATATTCCAAAGCCAACCTGGTGCATGAGCAGATAAAACCCAGGTTCC
CAGACTATGATGAAAAACAGTTTCTGCCATCGTTGGACGTACAGGCCCC
TGATTGTCAGTAGCGCACACGTCCAGCTTCCTCTTCGTCGAATCGTTCT
GAACCGGCGACGGCTTTGTAAACCAAGTCAAACCTGGGGGTGCCGATTG
TGTTCTGTCTGCTGAATTGTCGCTCACAGCCTAACTGCGGAACCACA
  1 ATGCTGTCTTCGCTGTGCATGATGGCCGCGATCTTTGGCTTTCCT
     M  L  S  S  L  C  M  M  A  A  I  F  G  F  P
 46 CTGAGAGAAGCGTTCGTCCTCCATCCCTCCCGAGAGGAGCCTGCA
     L  R  E  A  F  V  L  H  P  S  R  E  E  P  A
 91 GGAGCCATCTCAGACGTGCCGGTTGGCAAGGTGAACAGATGCCAG
     G  A  I  S  D  V  P  V  G  K  V  N  R  C  Q
136 GGGGAGTCACTTCAGGTCATAAAGCAGAAGCTGTTGGAGGCCCTG
     G  E  S  L  Q  V  I  K  Q  K  L  L  E  A  L
181 AACCTGGGTAGGGAGCCTCAGGTTTCCAGGACTGGACTCGGCAGG
     N  L  G  R  E  P  Q  V  S  R  T  G  L  G  R
226 TTTAGGGAGCAGTGGAAAGCGGTCTTAGGAGACACTGCCCACAGC
     F  R  E  Q  W  K  A  V  L  G  D  T  A  H  S
271 TCACCGAAGTCTCAGGAATCCACTGTGTTGGATACAACCTCCACA
     S  P  K  S  Q  E  S  T  V  L  D  T  T  S  T
316 CAAGAGTCCACTGGGACCCGAGATGAGACCAACAGTACAGGCTTG
     Q  E  S  T  G  T  R  D  E  T  N  S  T  G  L
361 CAGTGCTGCCAGCTGGCTTCACAGATCTTCATAAACGATCTTGGT
     Q  C  C  Q  L  A  S  Q  I  F  I  N  D  L  G
406 TGGGAGAACTGGATAATCTTTCCAGATACCTTCACCTACACCCAG
     W  E  N  W  I  I  F  P  D  T  F  T  Y  T  Q
451 TGTGCAGTCTGCGACCCCCACCTGGACCCAAAAGCTCCAAAGTGT
     C  A  V  C  D  P  H  L  D  P  K  A  P  K  C
496 CGAGCAAACAGCCCCCCTGAGCCAAACACCCCTTCAAAGTGCTGC
     R  A  N  S  P  P  E  P  N  T  P  S  K  C  C
541 CAGCCAACCTCCCATGTGATGGTGCCATTCTTCTACTTGGATGAG
     Q  P  T  S  H  V  M  V  P  F  F  Y  L  D  E
586 CTCAACACGCCAGTCATCTCCTCTGTGGCCCTGACCAACCAATGT
     L  N  T  P  V  I  S  S  V  A  L  T  N  Q  C
631 GGCTGCAAACCAGGATCATACATCCAGGATGCACAGAACTGA
     G  C  K  P  G  S  Y  I  Q  D  A  Q  N  *
```

Figure S1: Nucleotide and deduced amino acid sequences of A. japonica Gsdf. Start codon is shown in box.
