# Supplementary material for: Involvement of Transforming Growth Factor Beta Family Genes in Gonadal Differentiation in Japanese Eel, Anguilla japonica, According to Sex-Related Gene Expressions
Source: Cells. 2021 Nov 4;10(11):3007. doi: 10.3390/cells10113007 (PMC8616510; doi:10.3390/cells10113007)
Supplement: Supplementary file 1 [file cells-10-03007-s001.zip › Figure S2.pdf]

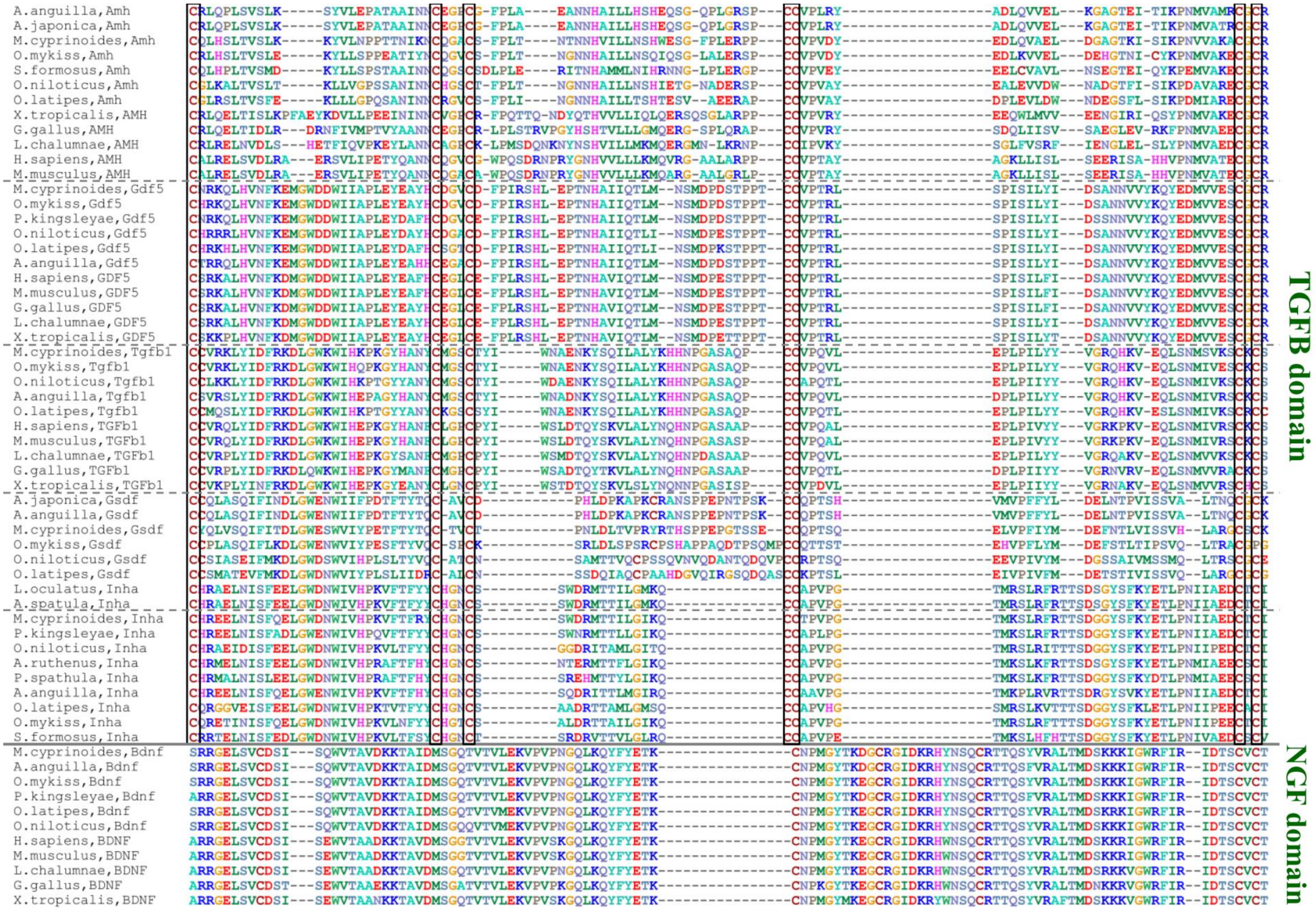

Figure S2: Alignment of 50 TGF-β domain and 11 NGF domain amino acid sequences used in the phylogeny analysis (in Figure 3). Multiple alignment of TGF-β domain (transforming growth factor-beta family) of Amh, Gdf5, Gsdf, Inha and Tgfb1; and NGF domain (nerve growth factor) sequences of Bdnf. Conserved amino acids of the cysteine knot of TGF-β domain are boxed.
